# The possible impact of escaped captive American mink (*Neogale vison*) on the population of feral mink in Denmark

**DOI:** 10.1371/journal.pone.0292609

**Published:** 2023-11-30

**Authors:** Tobias Astell Andersen, René Worup Rørbæk, Cino Pertoldi, Sussie Pagh

**Affiliations:** 1 Department of Chemistry and Bioscience, Aalborg University, Aalborg, Denmark; 2 Aalborg Zoo, Aalborg, Denmark; University of Seville Faculty of Biology: Universidad de Sevilla Facultad de Biologia, SPAIN

## Abstract

The Danish feral mink (Neogale vison) population can be divided into wild-born and captive-born mink newly escaped from a farm. The impact of the captive-born mink on the population of feral population is unknown. Captive-born mink has lately been estimated to make up 25–30% of the feral mink population. In December 2020 Danish mink farms were decommissioned until January 2023. The aim of this study was to: 1) Examine whether a supplementation of farmed mink into the feral mink population influence the population growth and extinction rate of the feral mink, 2) Investigate which parameters affect the viability in feral mink populations the most. The age and mortality of 351 mink and the reproduction of 85 adult females culled by hunters from 2019–2022 were determined in three distinct geographic areas of Denmark (Jutland, Zealand, and Bornholm). VORTEX simulations were used to model the population viability and to simulate the impact of a supplementation of captive-born mink into the wild population. Simulations show that changes by 30% in the parameters: fecundity, percentages of breeding females and mortality influenced population size influenced the final population size significantly. The initial population size and inbreeding did not affect the population growth significantly. The simulations showed that the mink population in the regions of Jutland and Zealand could go extinct within 15 to 30 years without any supplementation of captive-born mink to the feral population. The population on Bornholm will however remain stable with current levels of mortality and reproduction even without supplementation of captive mink.

## Introduction

All feral mink in Denmark are American mink (*Neogale vison*) that have escaped from fur farms. The feral mink population in Denmark consists of two subpopulations of mink; wild-born and captive-born mink [1]. The number of captive-born mink caught by trappers shows a substantial annual supplementation of farmed mink into the feral population. Originally, mink were introduced into Denmark during the establishment of the fur production in the 1920–30s [2]. Since then, mink have escaped farms into the wild, and a feral population has been established in Denmark, with the first documentation of feral mink in Denmark in the 1950’s [3, 4]. Previous Danish studies have found that 80% of the feral mink caught by trappers in the years 1998–2000 and 25–30% of mink caught during the winters of 2014–2018 were mink recently escaped from farms [1, 3].

Mink are semi-aquatic mammals belonging to the *Mustelidae* family. They can live near both freshwater and saltwater habitats. In Denmark, the population of mink has increased over time as reflected in the annual mink harvest which increased from approximately 1,000 in 1980 to nearly 8,000 mink in 2000. Recently, the trend in mink harvest has declined to 768 animals in the 2021/22 season [5].

Mink are regarded as a potential threat to the biological diversity, when introduced to an environment in Europe. Especially in bird protection areas mink may pose a threat to ground breeding bird colonies [6–9]. The competition between introduced mink and native red listed species, such as European polecat (*Mustela putorius*) and Eurasian otter (*Lutra lutra)*, has been discussed in England [6]. The disappearance of European water vole (*Arvicola terrestris*) in England is believed to be caused by introduced mink predation [6, 10, 11].

In Denmark, mink have been culled since 1970´s and they are trapped all year round in the wild because mink are listed as invasive according to the Bern Convention on the Conservation of European Wildlife and Natural Habitats recommendation nr. 77 [7].

Voluntary trappers are organized in more than 100 groups carrying out control (culls) of mink in natural habitats [12]. Before 2020 there were approximately one thousand farms housing 17 million mink in Denmark [4]. Most farms (985 in 2019) were in Jutland, where half of the municipalities had between 40 to 80 farms and the other half between 5 to 40 farms. On Zealand the number of farms were relatively low (39 in 2019). The density of farms on Zealand were between 0 to 40 farms per municipality. Bornholm had approximately 6 farms [13]

An outbreak of Coronavirus 2 (SARS-CoV-2) in Danish mink led the Ministry of Environment and Food of Denmark to decommission all Danish mink farm by December 30^th^ 2021 until January 2023. At present only 13 farms have been reopened, all of which are in Jutland [13].

The continual escape of captive-born mink have been suggested to prevent the establishment of a feral mink population, due to lower fitness found in the escaped captive-born mink, which may decrease the feral minks’ genetic adaptation to the wild [14–16]. On the contrary, a continuous supplementation of farmed mink into the feral mink population may prevent the extinction of feral mink population if escaped captive mink act as a supplementation to the wild born population.

To examine a potential extinction of a species a Population Viability Analysis (PVA) can be used. Population viability analysis is a method for the risk assessment of a species’ probability of extinction [14, 17]. The main advantage of PVA is the possibility to achieve an understanding of factors influencing the population development of species based on fecundity and mortality of the species [16].

The aim of this study is to examine the current population viability of feral mink in Denmark with and without the theoretical supplementaion of farmed mink. The research questions are:

1. Examine whether an supplementation of escaped captive-born mink support and help to maintain the feral mink population?
2. Investigate which parameters (fecundity, percent breeding females, number of lethal equivalents, initial population size and mortality) have the largest effect on the viability of the feral mink population.

## Materials and methods

### Study area

The study area includes three different regions of Denmark, Jutland, Zealand and Bornholm. Jutland is the Danish peninsula covering an area of 29,775 km^2^, bordered by Germany to the south. The northernmost point of Jutland is located at 57°43′N/10°37′E. Zealand covers 7,031 km^2^ while Bornholm extends 588.36 km^2^. Denmark is characterized by flat, arable land and sandy coasts, low elevation, and a mild coastal temperate climate. There are many small freshwater lakes, ponds and streams in every region of Denmark creating ample habitable niches for mink. Around 70% of the country comprises intensively human modified agricultural land. Bornholm differs geologically and in natural conditions from the rest of Denmark by having rockier ground similar to Swedish nature and having no native predators. Though the two largest Danish regions (Jutland and Zealand) are isolated by the Great Belt, the mink populations live under similar natural conditions except from otters being more widespread in Jutland than in Zealand. Denmark has two natural barriers for mink populations in Denmark, the Great Belt Strait separating Jutland and Funen from Zealand and the Baltic Sea separating Bornholm from mainland Denmark (Jutland, Funen and Zealand). These barriers act as natural separators of mink populations between Jutland, Zealand and Bornholm, preventing them from mixing with each other, as the distance a mink can swim does not exceed five kilometres [18]. Funen was not included in this study, as only one mink was received from Funen.

### Ethics

This study was supported by the Danish Ministry of Environment (Authorization J.nr. 2021–49772).

### Collection of specimens

Specimens for this study were delivered to the University of Aalborg or The Technical University of Denmark, Lyngby from three regions of Denmark during the years 2019–2022.

The study was carried out as part of the control program for mink and the surveillance of wildlife diseases under the Danish Ministry of Environment. All present national, regional and institutional guidelines for the care and use of the animals were followed. The study was compiled within the frame word of current Danish law.

Most mink were caught in traps either in kill-traps or in live catch traps and then put down by trappers. Few mink were shot or were road kill. The trappers submitted information on date, capture location, and information about when and where the mink was put down. Specimens were kept at -20°C until the necropsy. Necropsy data collected include sex, body length (from tip of nose to start of tail, tail length (start of tail to last vertebra), and fur colour. A recent study has shown that captive-born mink older than 4 months can be separated from wild-born mink using body length, and mink in this study were divided accordingly [1]. Males with a body length below 43 cm and females with a body length below 39 cm were considered to be wild born following the method used in former studies [1].

### Life tables and fecundity calculations

The life tables were made by grouping the mink from the three populations (Jutland, Zealand and Bornholm) into different age categories. The categories being <1 year old, >1 year old, >2 years old, >3 years old and older than 4 years. The mortality rate q(x) in the different age groups was calculated by dividing the number of individuals that had died within the age group with the number of individuals in the beginning of the age group (Table 1). The initial population in each region was calculated from fecundity of the population in question.

**Table 1:** The age of the collected American mink (*Neogale vison*) from Jutland, Zealand, and Bornholm (n = 351), the mean mortality rate of the populations both for the first year and the following years. The mean litter size, fecundity, and percentage of adult breeding females from Jutland, Zealand and Bornholm are shown. Generation zero is shown with a * and is the theoretical amount of mink born based on the gamebag records.

Region	Jutland	Zealand	Bornholm
Mink (n)	62	134	155
Generation 0	33*	79*	126*
<1 (n)	29	51	68
>1 (n)	25	49	61
>2 (n)	5	31	24
>3 (n)	2	2	2
>4 (n)	1	1	0
Mean mortality rate (<1)	0.24	0.38	0.46
Mean mortality rate (>1)	0.59	0.58	0.4
Litter size	6	4.7	5.8
Fecundity	2	1.9	2.9
% Adult breeding females	33%	42%	50%

### Estimation of input parameters for VORTEX

VORTEX was used for population simulations [19]. The program is a well-known and recognized method of simulating deterministic forces, environmental and demographic stochastic events and genetic drift in populations and it is used to perform a PVA [20–22]. The software requires input parameters to run a PVA. These parameters are carrying capacity, mortality rate, fecundity, proportion of adult breeding females and proportion of males in the breeding pool, age of maturity, oldest individual, number of lethal equivalents (inbreeding depression), sex ratio, and harvest and supplementation in case of a decrease or increase to the population due to trappers or captive breeding respectively [17]. Extinction was defined as when one sex remained in the simulations [17]. PVA is a method mostly used for conservation of endangered species. However the study aims to use a PVA model for estimation the extinction of non-native species, mink in the study [17].

### Demographic parameters and carrying capacity

Demographic parameters and carrying capacity for the three populations in Denmark (Jutland, Zealand, and Bornholm) were estimated based on literature and reproductive productivity tests. General demographic and genetic parameters for mink are the following parameters:

1. Number of lethal equivalents of 6.29 [23],
2. The age of maturity, which was set at one year,
3. Maximum lifespan of five,
4. A polygynous reproductive system,
5. The proportion of males in the breeding pool, which was set at 33% for all three populations due to the breeding strategy and territoriality of mink. Males may have limited access to females during the mating period [24],
6. Carrying capacity, which was set at 30,000 for Jutland, 7,100 for Zealand and 500 for Bornholm based on habitat availability calculated by Hammershøj [16].

Population specific demographic parameters such as mortality rates, fecundity, proportion of females in the breeding pool were estimated from the mink culled from the three regions; Jutland, Zealand and Bornholm.

The proportion of females, fecundity and mortality rates were calculated from the life tables of the culled mink. This means that hunting pressure was part of the mortality rates, hence harvest was not included in the simulations. The initial population size of the feral mink population is unknown as there is no population inventory of Danish mink. Assuming that the main mortality of mink in Denmark is culling and that the initial population size for the coming year in a stable population should be at least the number of the culled individuals in the previous year, the initial population size was initiated from two scenarios:

1. A scenario using the most recent data available from the NGBR from the hunting season 2021/2022.
2. A scenario using the data from the year with the maximum Game Bag Records for each of the three regions Denmark (Jutland year 1999, Zealand year 2013 and Bornholm year 2019) [5].

These scenarios will be called the “recent” and “maximum” hereafter and they were made to predict the current state of affairs for feral mink compared to before the closing of the mink farms.

### Simulations

The main simulation was a supplementation test including a sensitivity test. All the simulations were run with 100 iterations and over a 100-year time period. The supplementation simulations were conducted with the demographic parameters and carrying capacity and analysed by the values of the mean stochastic growth rate (Stoch-r), mean population size (N-all) and the mean time of extinction (mean TE). The parameter supplementation varies between an annual supplementation with 0, 10, 20, 40, 100 and “influx” individuals, in all three populations. The “influx” was an amount based on 30% of the initial population size, since approximately 30% of the mink of the mink caught by trappers in the wild were captive-born mink, and must be the supplementation from farms [1]. This was done to simulate the effects of mink escaping from farms on the free-ranging population with different amounts of escaping mink. To test the sensitivity of the parameters to annual changes, standard parameters were tested with a ± 20% and a ± 30% change in the following demographic parameters: fecundity, percentage of adult breeding females, mortality, number of lethal equivalents and initial population size in respect of the initial starting values for the different populations and analysed by the values of the mean Stoch-r, N-all and mean TE.

### Statistical analysis

Mann-Whitney U tests were used to test whether the median of growth rates were significantly impacted by parameters in the sensitivity tests since the data were not normally distributed [25].

## Results

In total, 375 mink were collected. All were sexed (172 males and 203 females). Of these, 80 were from Jutland (22 males and 58 females), 137 from Zealand (58 males and 79 females) and 158 from Bornholm (92 males and 66 females). The uteri of 85 adult females (15 from Jutland, 36 from Zealand and 34 from Bornholm) were analysed.

### Life and fecundity table

Of the 375 collected mink, 351 were aged to calculate the juvenile/adult ratio (J/A) for Jutland: 0.88 (29/33), Zealand: 0.61 (51/83); and Bornholm; 0.78 (68/87). The overall (J/A) ratio was 0.70 (148/203).

Only two mink were older than four years in this study both being captive-born from Bornholm and Jutland respectively. The oldest mink was estimated to be almost five years old, due to it having four dental lines in the canine tooth, and it was culled in March.

The mean mortality rate for the first year was 0.24, 0.38 and 0.46 for Jutland, Zealand, and Bornholm respectively (Table 1). The mean mortality for mink after the first year was estimated at 0.40 in Jutland, while it was 0.37 in Zealand and estimated at 0.30 on Bornholm (Table 1). The mean litter size was 6, 4.7 and 5.8 for Jutland, Zealand and Bornholm, respectively. The percentage of adult breeding females in the populations was 33% in Jutland, 42% Zealand and 50% in Bornholm. Hence, fecundity was 2.0, 1.9 and 2.9 respectively (Table 1).

### Sensitivity of parameters

**Fig 1:**
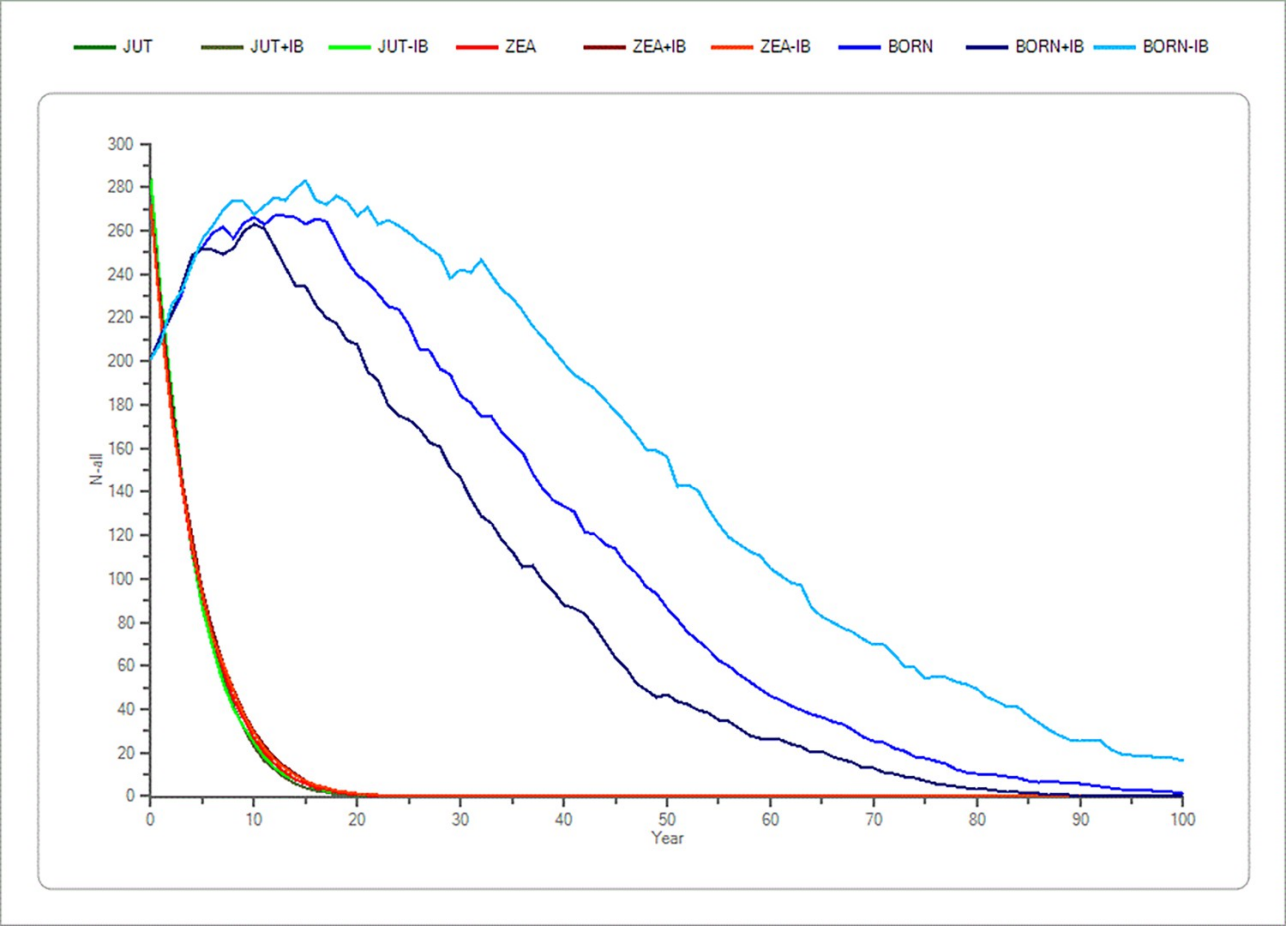
Sensitivity test of lethal equivalents (IB) in the three populations (Jutland, Zealand and Bornholm). The average development based on the “recent” scenario’s basic parameters of the lethal equivalents (IB) in the three populations of mink (*Mustela vison*) plus a ±20% change in IB. Bornholm is shown with blue colours. Zealand is shown with red colours and Jutland is shown with green colours. The x-axis shows the years, and the y-axis shows the amount of mink (n).

**Fig 2:**
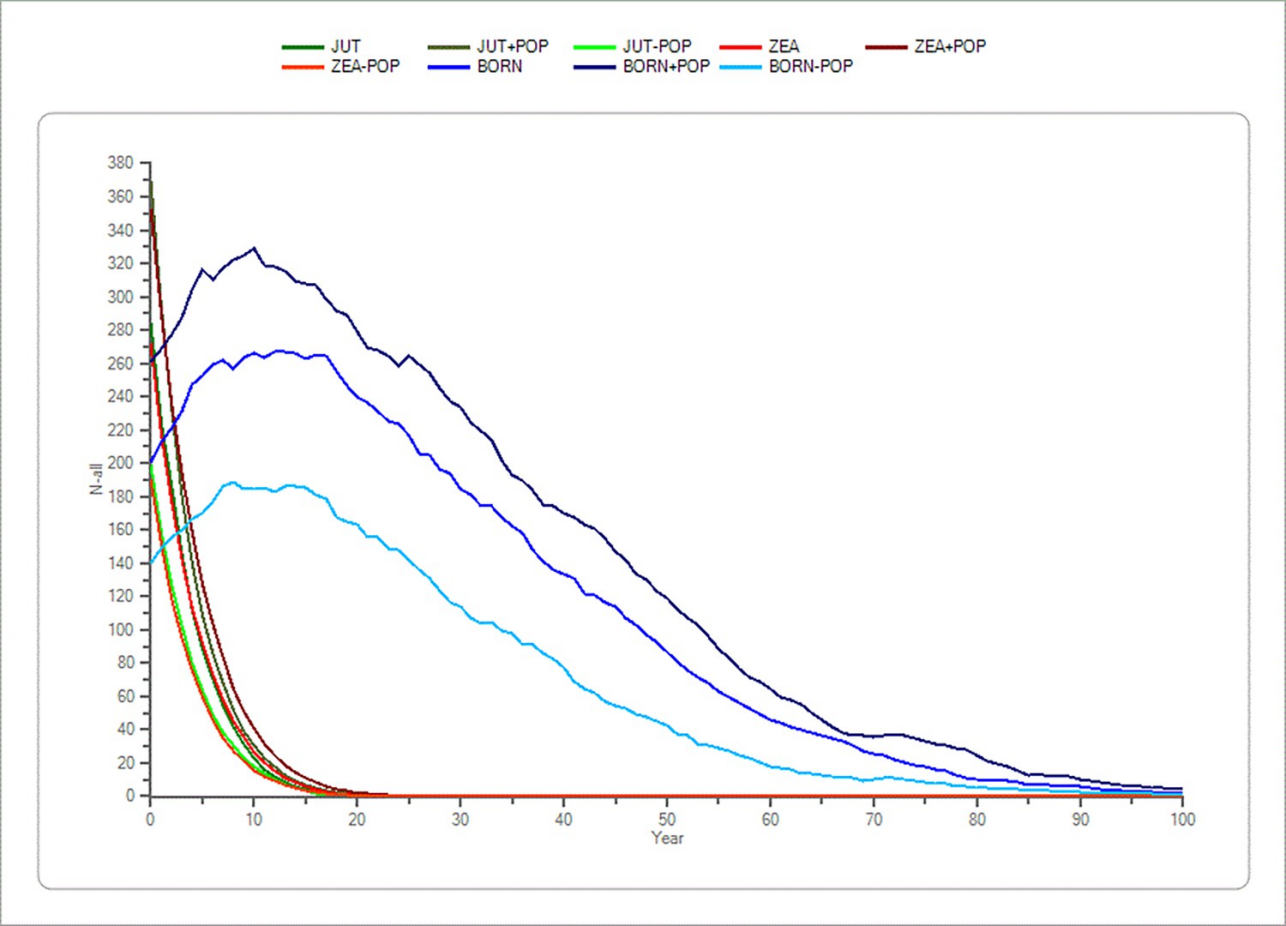
Sensitivity test of initial population size (POP) in the three populations (Jutland, Zealand and Bornholm). The average development based on the “recent” scenario’s basic parameters of the initial population size (POP) in the three mink (*Mustela vison*) populations plus a ± 20% change in POP. Bornholm is shown with blue colours. Zealand is shown with red colours and Jutland is shown with green colours. The x-axis shows the years, and the y-axis shows the amount of mink (n).

The parameters number of lethal equivalents and initial population size’s p-values never showed a significant difference in the mean TE compared to the standard simulations (p > 0.05) in the Jutland and the Zealand population (S4 and S5 Tables in S1 File), only on Bornholm with a ± 30% change a significant difference in the outcome of simulations being noticed in the mean TE. These parameters produced only minor changes in the outcome of simulations compared to the standard simulations. This indicates that inbreeding depression and initial population size did not have a significant impact on the results (S4 and S5 Tables in S1 File and Figs 1 and 2).

In Jutland and Zealand, the parameters fecundity, proportion of adult breeding females and mortality, produced no significant difference with a ± 20% change from the standard parameters (Table 2).

**Table 2:** The values of the mean stochastic growth rate (Stoch-r), mean population size (N-all) and the mean time of extinction (Mean TE) for all the sensitivity test simulations (Inbreeding, initial population size, proportion of adult breeding females, fecundity and mortality) done for all three populations (Jutland, Zealand and Bornholm) with the 20% sensitivity tests.

Sensitivity testing index 20%	Jutland			Zealand			Bornholm		
	Stoch-r	N-all	Mean TE	Stoch-r	N-all	Mean TE	Stoch-r	N-all	Mean TE
Standard	-0.3003	0	15.3	-0.2709	0	16.9	-0.0607	0.81	59.1
Lethal equivalents +20%	-0.2969	0	15.3	-0.2781	0	16.4	-0.0739	0.24	52.7
Lethal equivalents -20%	-0.2913	0	15.8	-0.2665	0	17.3	-0.0548	6.02	59.7
Fecundity +20%	-0.2067	0	21.6	-0.2142	0	21	0.0426	287.71	87.2
Fecundity -20%	-0.3546	0	12.9	-0.3447	0	13.3	-0.1498	0	28
Adult breeding females +20%	-0.2389	0	19.3	-0.214	0	21.1	0.0471	300.76	72.3
Adult breeding females -20%	-0.3861	0	11.9	-0.3367	0	13.4	-0.1512	0	27.8
Initial population size +20%	-0.2959	0	16.2	-0.2804	0	17.1	-0.057	4.63	63.1
Initial population size -20%	-0.3013	0	14.4	-0.2718	0	16	-0.0669	2.45	52.4
Mortality +20%	-0.3964	0	11.9	-0.3836	0	12.3	-0.1738	0	24.2
Mortality -20%	-0.217	0	21	-0.1843	0	24.5	0.0888	448.81	0

**Fig 3:**
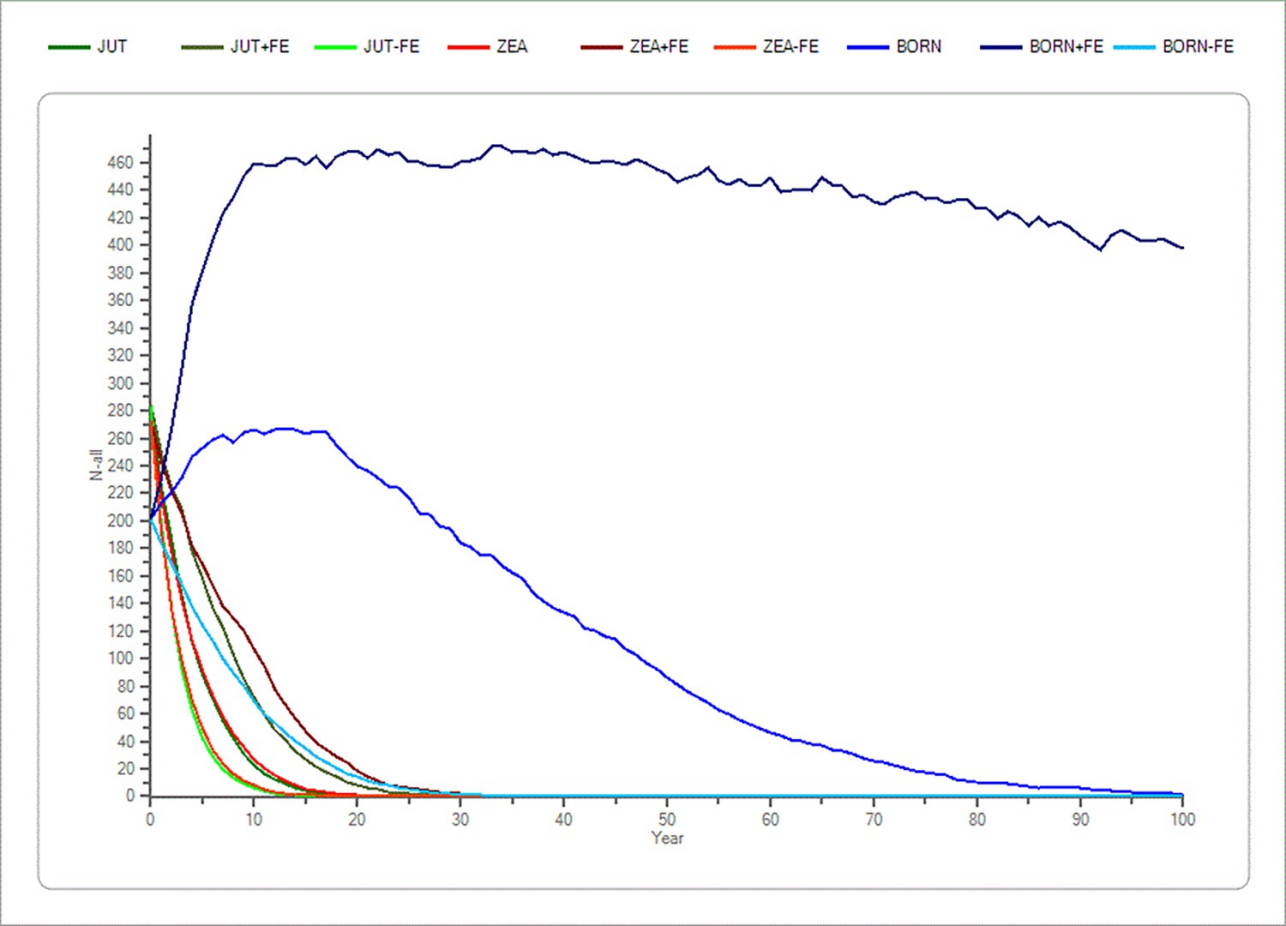
Sensitivity test of fecundity (FE) in the three populations (Jutland, Zealand and Bornholm). The average development based on the “recent” scenario’s basic parameters of the fecundity (FE) in the three populations plus a ± 20% change in FE. Bornholm is shown with blue colours. Zealand is shown with red colours and Jutland is shown with green colours. The x-axis shows the years, and the y-axis shows the amount of mink (n).

With an increase of 30% in fecundity, the Zealand population produced a significant difference in the mean TE, while an increase of 30% in percentage adult breeding females produced significant differences in the mean TE of the mink population in Jutland and on Zealand (Fig 3 and Table 2).

With a change of ± 30% in the number of adult breeding females in Jutland and on Zealand, the mean TE of the two populations ranged between 12.0 and 20.9 and 12.7 to 24.2 years, respectively (Table 3).

**Table 3:** The values of the mean stochastic growth rate (Stoch-r), mean population size (N-all) and the mean time of extinction (Mean TE) for all the sensitivity test simulations (inbreeding, initial population size, proportion of adult breeding females, fecundity and mortality) done for all three populations (Jutland, Zealand and Bornholm) with the 30% sensitivity tests.

Sensitivity testing index 30%	Jutland			Zealand			Bornholm		
	Stoch-r	N-all	Mean TE	Stoch-r	N-all	Mean TE	Stoch-r	N-all	Mean TE
Standard	-0.3071	0	15.1	-0.2765	0	16.4	-0.0592	1.53	59.9
Lethal equivalents +30%	-0.3079	0	14.9	-0.271	0	16.7	-0.0749	0.03	52
Lethal equivalents -30%	-0.2865	0	16.1	-0.2615	0	17.3	-0.0409	16.21	66.5
Fecundity +30%	-0.2167	0	20.9	-0.1864	0	24.2	0.0782	398.19	0
Fecundity -30%	-0.3981	0	12	-0.3686	0	12.7	-0.1769	0	23.5
Adult breeding females +30%	-0.2105	0	21.6	-0.1858	0	24	0.0901	445.76	0
Adult breeding females -30%	-0.42	0	11.4	-0.3843	0	11.9	-0.1856	0	22.6
Initial population size +30%	-0.2927	0	16.4	-0.2666	0	17.9	-0.0562	4.26	62.8
Initial population size -30%	-0.278	0	14.6	-0.2911	0	14.5	-0.071	0.69	50.3
Mortality +30%	-0.4589	0	10.4	-0.4326	0	10.8	-0.2458	0	17.4
Mortality -30%	-0.1713	0	26.7	-0.1315	0	33.7	0.1442	487.13	0

**Fig 4:**
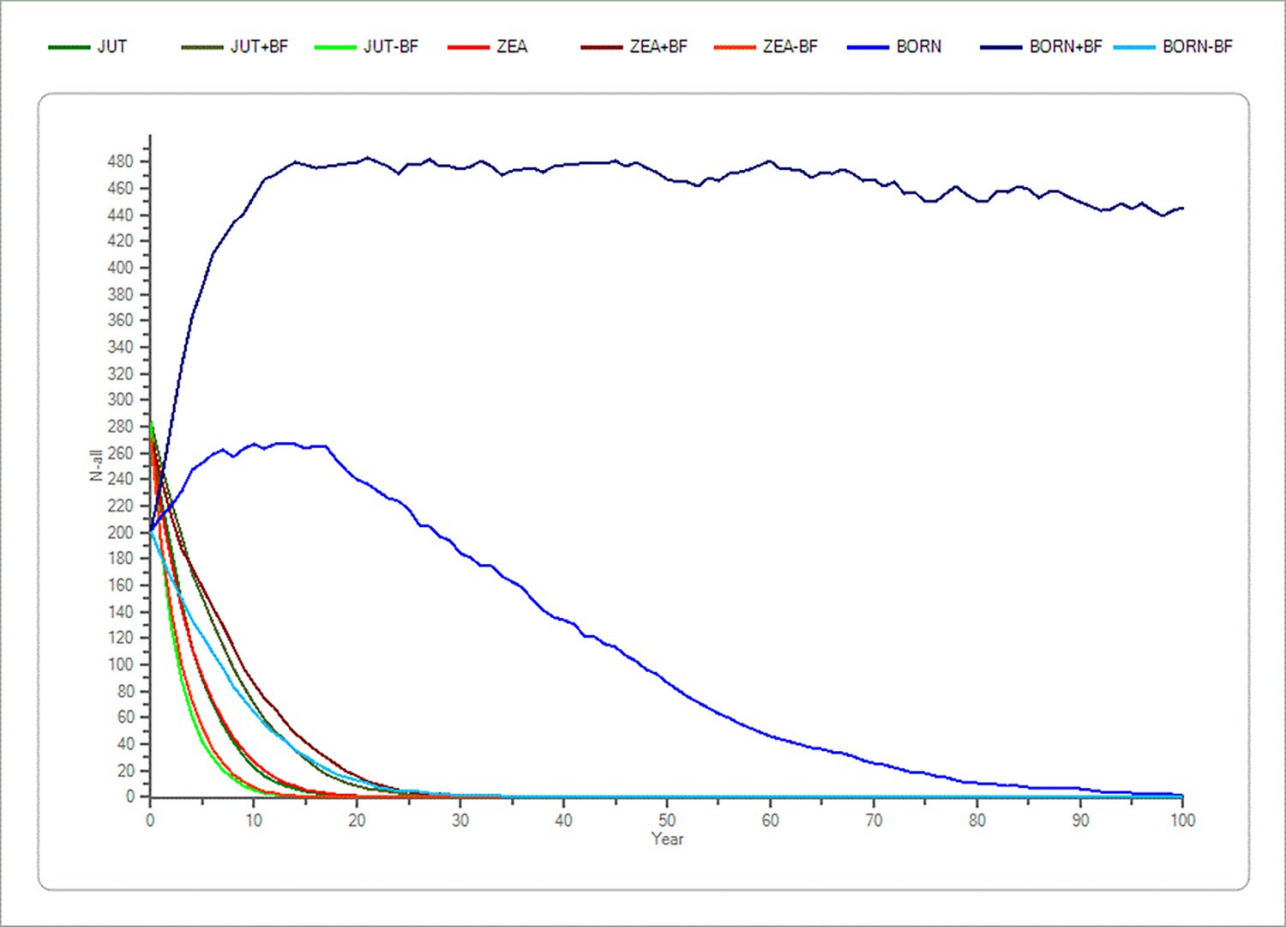
Sensitivity test of adult breeding females (BF) in the three populations (Jutland, Zealand and Bornholm). The average development based on the “recent” scenario’s basic parameters of the percentage of adult breeding females (BF) in the three populations plus a ± 20% change in BF. Bornholm is shown with blue colours. Zealand is shown with red colours, and Jutland is shown with green colours. The x-axis shows the years, and the y-axis shows the amount of mink (n).

Bornholm differed from the two other populations as a change in the standard parameters ± 20% and ± 30% showed significant differences from the standard simulations (Tables 2 and 3). A decrease in the percent adult breeding females of 20% and 30% showed a difference in mean TE of 27.8% and 23.5%, and an increase in the percent adult breeding females of 20% and 30% postponed extinction time to 72.3 years and no extinction within 100 years, respectively (Fig 4 and Tables 2 and 3).

**Fig 5:**
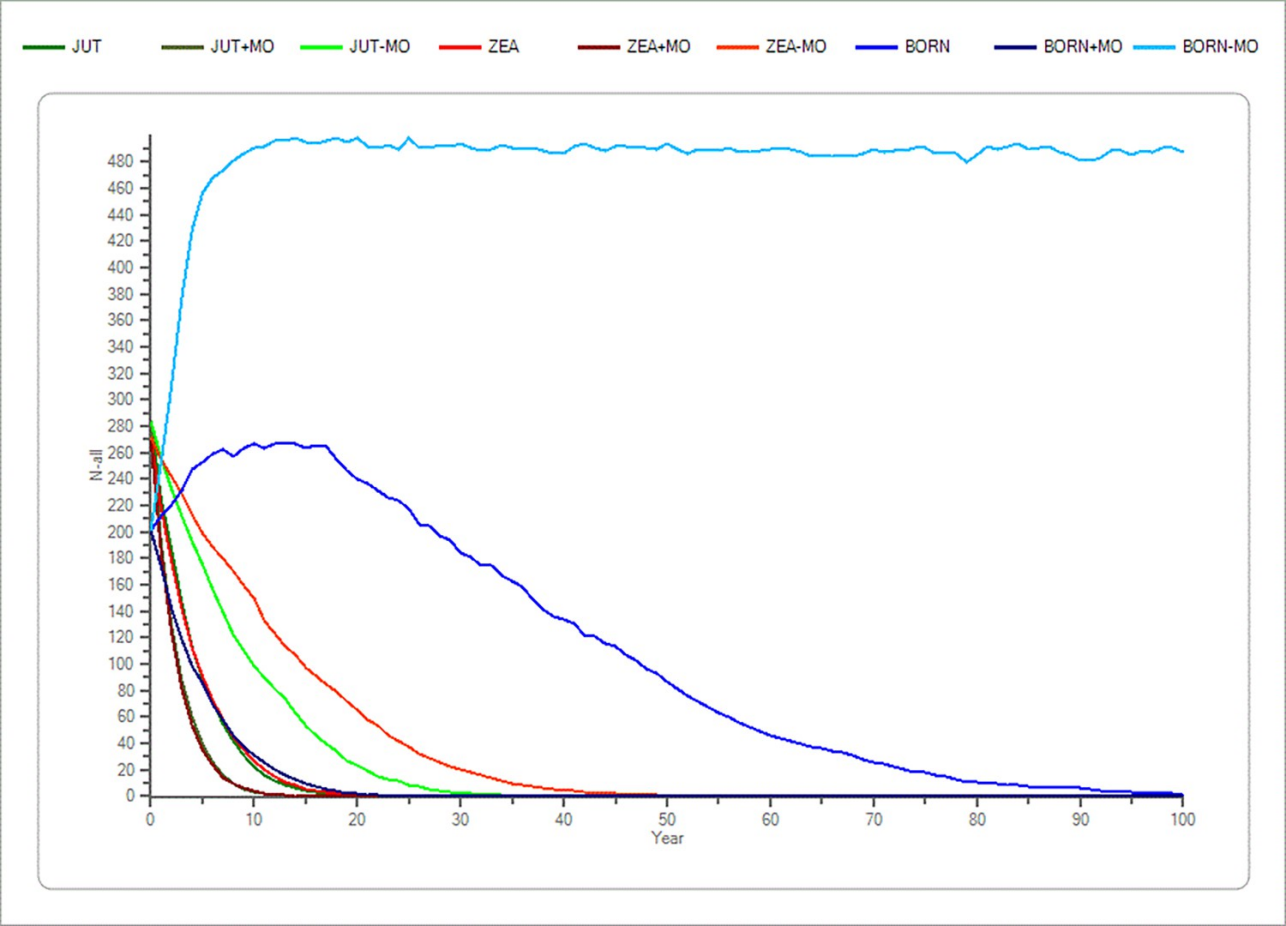
Sensitivity test of mortality (MO) in the three populations (Jutland, Zealand and Bornholm). The average development based on the “recent” scenario’s basic parameters of the mortality (MO) in the three populations plus a ±20% change in MO. Bornholm is shown with blue colours. Zealand is shown with red colours, and Jutland is shown with green colours. The x-axis shows the years, and the y-axis shows the amount of mink (n).

A change in mortality showed significant changes in the extinction rate in the three populations. A decrease of 30% in mortality postponed the extinction in Jutland from 15.1 years to 26.7 years, and for Zealand from 16.4 to 33.7, while Bornholm went from 59.9 to not going extinct with a decreased mortality (Fig 5 and Tables 2 and 3).

### Population simulations with and without supplementation of farmed mink

**Fig 6:**
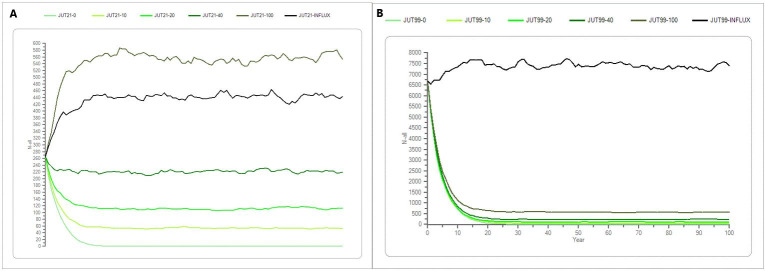
A and B the average development of the mink population in Jutland. (A) shows the recent scenario in Jutland with a supplementation of 0, 10, 20, 40 and 100 individuals per year, in addition of an influx supplementation of 20% of the initial population size, which was 80 and 1486 individuals yearly in the recent and maximum scenario, respectively. The different shades of green symbolize the different supplementation scenarios. The x-axis shows the years, and the y-axis shows the number of mink (n). (B) is the same but for the maximum scenario.

The simulations show that the stochastic growth rate (Stoch-r) in the Jutland mink population is negative, and that the population will go extinct without a supplementation of farmed mink within about 15 to 30 years (Table 4 and Fig 6A). With the “recent” scenario (initial population size of 266 individuals) the population will go extinct within approximately 15 years and with a “maximum” scenario (initial population size of 6695 individuals) the population will go extinct within 29 years (Table 5 and Fig 6B).

**Table 4:** The values of the mean stochastic growth rate (Stoch-r), mean population size (N-all) and the mean time of extinction (Mean TE) for all three populations (Jutland, Zealand and Bornholm) and supplementation simulations (0,10, 20,40,100 and “influx”) with the most recent data from 2021.

Supplementation index (recent)	Jutland			Zealand			Bornholm		
	Stoch-r	N-all	Mean TE	Stoch-r	N-all	Mean TE	Stoch-r	N-all	Mean TE
0	-0.3003	0	15.1	-0.2807	0	16.4	-0.0669	0.46	55.9
10	-0.0166	56.14	0	-0.0158	58.42	0	0.0393	418.69	0
20	-0.0098	109.75	0	-0.0011	249.15	0	0.0712	450.08	0
40	-0.0026	225.18	0	-0.0009	255.19	0	0.1143	486.66	0
100	0.0071	595	0	0.0082	630.73	0	0.2299	499.06	0
Influx	0.0008	318.18	0	0.0017	329.82	0	0.1179	490.75	0

**Table 5:** The values of the mean stochastic growth rate (Stoch-r), mean population size (N-all) and the mean time of extinction (Mean TE) for all three populations (Jutland, Zealand and Bornholm) and supplementation simulations (0,10, 20,40,100 and “influx”) Table 5 is from the year with the highest number of mink found in the region (1999, 2013,2019).

Supplementation index (Before)	Jutland			Zealand			Bornholm		
	Stoch-r	N-all	Mean TE	Stoch-r	N-all	Mean TE	Stoch-r	N-all	Mean TE
0	-0,2763	0,00	28,7	-0,2637	0,00	22,1	-0,0543	3,38	64,9
10	-0,0496	53,91	0,0	-0,0292	59,03	0,0	0,0373	413,48	0,0
20	-0,0426	108,67	0,0	-0,0220	119,66	0,0	0,0693	463,23	0,0
40	-0,0350	229,37	0,0	-0,0149	242,49	0,0	0,1160	488,40	0,0
100	-0,0262	555,03	0,0	-0,0051	645,09	0,0	0,2294	500,15	0,0
Influx	0,0010	8357,41	0,0	0,0022	1328,68	0,0	0,1452	493,41	0,0

Whereas, with a yearly supplementaion of only 10, 20, 40, individuals corresponding to approximately 4%, 7% and 14% of the initial population size, respectively, the Jutland mink population will in the “recent” scenario undergo a steep decline but maintain at a lower level (Table 4 and Fig 6A).

In the “recent” scenario in Jutland, a supplementation of approximately 30% of the Stoch-r is zero meaning that the initial population will remain stable, whereas a supplementation of 35% means the Stoch-r is positive, and the mink population will increase slightly, and the population will stabilize around twice the initial population size in 10 years. Parallel to the “recent” scenario the Stoch-r is negative in the “maximum” scenario with a supplementation below 30% of the initial population size, but remains low i.e., the population will remain at a low population size but not go extinct. A yearly influx above 30% the Stoch-r is positive, and the population will stabilize at a higher population size after approximately 10 years (Table 5 and Fig 6B).

**Fig 7:**
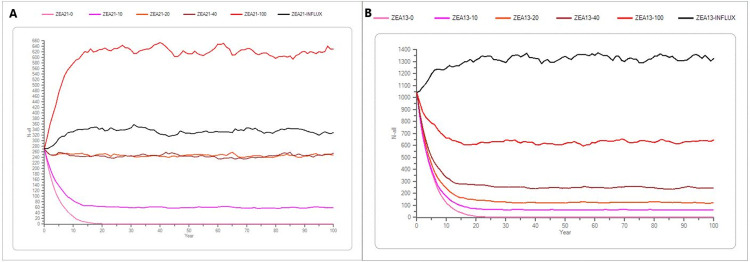
A and B The average development of the mink population on Zealand. (A) is the recent scenario for Zealand with a supplementation of 0, 10, 20, 40 and 100 individuals per year, in addition of an influx supplementation of 20% of the initial population size, which was 82 and 210 individuals yearly in the recent and maximum scenarios, respectively. The different shades of red symbolize the different supplementation scenarios. The x-axis shows the years, and the y-axis shows the number of mink (n). (B) shows the same but for the the maximum scenario.

Simulations of the Zealand population with both a “maximum” scenario (of 1044 individuals) and a “recent” scenario (initial population of 273) the Stoch-r is negative without influx and the population will go extinct after approximately 20 years, whereas a yearly influx of 10, 20 and 40 individuals the Stoch-r is slightly negative, and the mink population remains at a lower population size but does not go extinct (Tables 4 and 5 and Fig 7A and 7B).

In both scenarios of initial population, the growth rate is positive when the yearly supplementation is more than 20% of the initial population size, and the population will reach a higher population size in 15 to 20 years (Tables 4 and 5 and Fig 7A and 7B).

**Fig 8:**
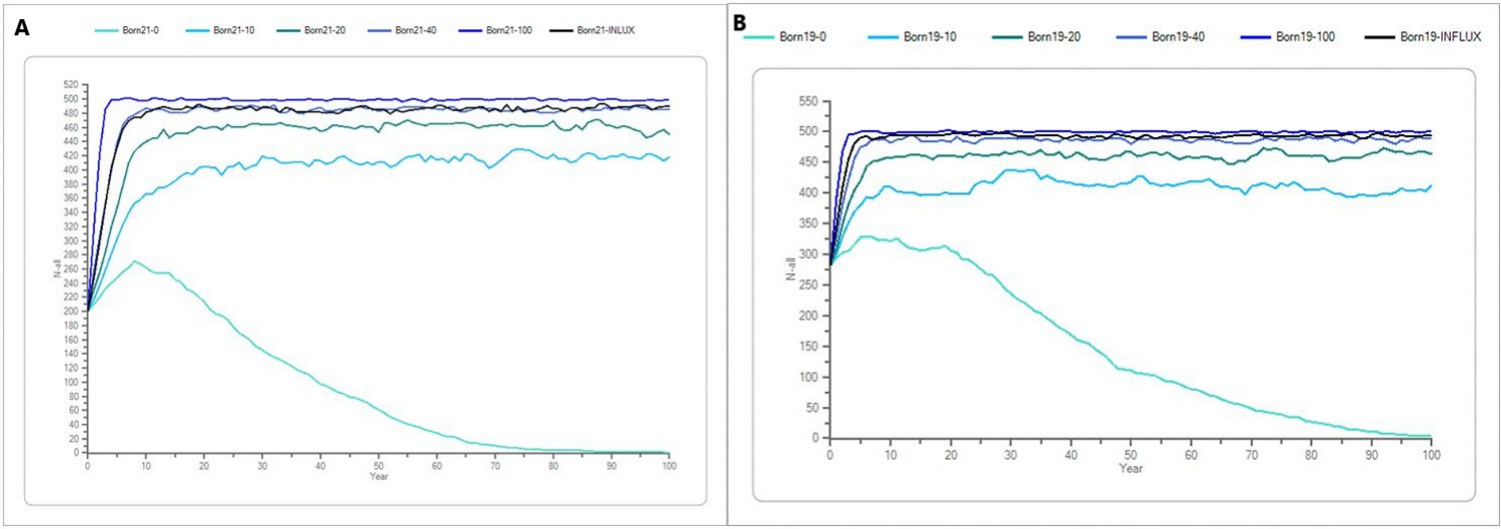
A and B the average development of the mink population on Bornholm. (A) shows the recent scenario for Bornholm with a supplementation of 0, 10, 20, 40 and 100 individuals per year, in addition to an “influx” supplementation of 30% of the initial population size, which was 40 and 56 individuals yearly in the recent and maximum scenarios, respectively. The different shades of blue symbolize the different supplementation scenarios. The x-axis shows the years, and the y-axis shows the number of mink (n). (B) shows the same but the maximum scenario for Bornholm.

The mink population on Bornholm differs from the populations in Jutland and Zealand by not going extinct the next 65 years in neither the “recent” (initial population size of 201 individuals nor the “maximum” scenario (initial population size of 282). In both scenarios which are similar, a supplementation of 10 individuals corresponding to 5% of the initial population per year the Stoch-r is positive, and the mink populations will increase and stabilize at a higher population size. With more than 30% supplementation, the population will increase and reach carrying capacity in five years (Tables 4 and 5 and Fig 8A and 8B).

## Discussion

### Vortex as a simulation program

Simulation programs such as VORTEX will only show a realistic population scenario with realistic parameters. In this study, simulations were based partly on known parameters such as fecundity, proportion of adult breeding females and mortality in the Danish feral population and unknown parameters such as initial population size and lethal equivalents. PVA is a debated method since criticism of the difficulty in collecting or assessing accurate input data and defining appropriate length of time for the simulations [14]. Former studies and tests have, however shown accurate prediction using population viability analyses [20–22].

### Sensitivity test

Simulations showed that changes in reproduction parameters less than 30% will only have a minor influence on the population simulations and the concluding population status. Higher and lower reproduction and mortality only influenced population simulations significantly when the two parameters exceeded or were lower than 30% of the standard simulation. Therefore, slight yearly variations in reproduction and mortality parameters are not considered to change results considerably.

The initial population size in the three populations was based on the number of culled mink during the hunting season 2021/2022 (Bornholm 201, Zealand 273, and Jutland 266) and years with maximum NHGB with the highest number of mink culled in the three Danish regions (Bornholm 282, Zealand 1044, Jutland 6695) [5, 16, 26]. The actual population size of mink is however not known. Nevertheless, the sensitivity simulations showed that neither initial population size nor the inbreeding parameter had significant influence on the growth rate or extinction time of the mink populations. Hence, the true population size of mink was not considered essential for the simulations, to simulate changes in the population due to influx of captive-born mink.

Fecundity was relatively low compared to litter size and an increase in fecundity of 30% was only significant for Bornholm and Zealand, not for Jutland. An increase in percent of adult breeding females delayed extinction time in all three populations (Fig 4). Also, the parameter mortality rate showed some impact on the extinction time. A decrease in mortality postponed extinction time, indicating that hunting pressure has an effect on the population, especially on Bornholm.

### Population viability analysis

The parameters fecundity, percent of adult females breeding and mortality in this study are based on demography of culled mink in Jutland, Zealand and Bornholm. The parameters are considered solid, except for Jutland from where less than 100 mink were received. The low amount of mink received from Jutland could be due to different hunting pressures in the three regions or that mink are outcompeted by otters or foxes [27, 28].

Jutland has historically had the largest amount of culled mink [5].

The mean litter size in this study was found to range between 4.7 and 6 kits which is lower than that found in a previous study of Danish mink from 2018, where the mean litter size was found to be 7.6 kits [1]. The mean litter size of Danish mink in 2018 may however have been higher than normal, as mean litter size in other European studies has been found to range between 4–7 kits [20, 29–31]. The number of barren females was in both the previous Danish study and this study relatively high approximately 50% compared to mink from Scotland, where 81% of the females were found to reproduce [15]. This may rely on a relatively high hunting pressure reducing the presence of adult and sexually mature females.

The mean mortality rate was slightly lower in this study (between 0.40 and 0.58) than compared to 2018, where the mean mortality was found to be 0.69 [1]. Mortality was higher within the first year, than in one-year-old and in two-year-old individuals. Mortality and reproduction may vary between years due to climate and the availability of basic diet for mink like rodents, amphibians, birds and fish [3].

The impact of a supplementation has in a previous study been expected to reduce the possibility of the populations going extinct, since only a small amount of unrelated mink supplemented to the populations could maintain the populations genetic variability and not go extinct due to inbreeding [14]. It has also been suggested that captive-born mink could have a negative impact on the wild-born populations since they are poorly adapted to the wild and may bring a flow of maladapted genotypes to the wild-born populations and prevent an adaptation to the wild in the wild-born populations [14, 16]. The latter has not been confirmed by nighter the simulations nor by the NGBR as years with a high percentage of escaped farm mink in the feral populations e.g. 1998–200 were also the years with maximum NGBR yield reflecting a high population of mink. Assuming that farmed mink are able to survive in nature despite their larger body size and no beforehand training in catching prey, the simulations show that with the exception of Bornholm, the Danish mink population will become extinct without supplementation from farm mink (Figs 6A, 7A and 8A).

### The Bornholm population seemed more resilient than the Jutland and Zealand populations

The simulations of the mink population on Bornholm, considered to be a more resilient population than those in Jutland and Zealand without supplementation with farmed mink (Fig 8A and 8B). The number of captive-born mink among the culled mink on Bornholm has been found to be less than 1% [4]. According to the game bag records, the mink population on Bornholm has been increasing slightly by approximately 15% per year since 2019 [5] which is in agreement with the predictions (Fig 6A). The maintained population of mink on Bornholm without influx may be due to the ample prey for mink on Bornholm where mink are the only wild terrestrial predator [4]. The absence of other terrestrial predators on Bornholm, especially the absence of red fox (*Vulpes vulpes*) and the Eurasian otter may give the mink more both terrestrial and aquatic prey options than in the mainland of Denmark, which could lead to a more resilient mink population on Bornholm [32, 33].

### The influence of mink farms on the Danish mink population

In the years before the closure of farms, the NGBR was decreasing by an average of 7% [5]. After the closure of the Danish mink farms, the number of culled mink decreased by approximately 50%, which support the results of the simulations that supplementation of captive mink into the wild, helps maintain the feral mink population in Denmark. The tightening of the rules on the enclosures of the farms around year 2000 also seemed to be reflected in a decrease in the mink population and a lower percent of farmed mink among culled mink. The number of mink culled in Denmark have been decreasing from 8000 in the 2000’s to approximately 1000 mink in 2016 [34].

The feral mink population in the regions of Jutland and Zealand may well depend on the supplementation of farmed mink, which has been between 25–30% before the closing of the mink farms [1]. The simulations and the game bag statistics of mink indicate that not only fecundity and mortality seriously affect population growth and sustainability, but that influx of escaped farm mink may have a decisive impact on the growth and maintenance of mink in Danish nature. The PVA seems to reflect the population trends of mink seen in the Danish NGBR and may therefore be reliable in estimating the extinction rate of other non-native species and for evaluation the parameters affecting the species the most [5]. Which means the PVA can be a viable tool to improve the management of invasive species, and conservation of biodiversity as the invasive species can affect the indigenous fauna community [35, 36].

## Conclusion

If escaped captive-born mink survive and reproduce in nature like wild-born mink, then captive-born mink have an impact on the feral mink population. The simulations show that with existing reproduction and mortality, the feral mink populations in Jutland and Zealand could decrease and go extinct within 15 to 30 years, due to the relative low fecundity compared to mortality in the feral Danish mink population, while the mink population on Bornholm will continue to persist for many years ahead.

The sensitivity tests showed that the most discriminating parameters in the simulations are percent of adult breeding females and mortality. Initial population size and inbreeding showed minor impact on the extinction time and were only significant in the mink simulations on Bornholm. This relatively fast extinction rate without supplementation into the Jutland and Zealand feral population indicates that the American mink may lose terrain in the competition with native predators. Results from Bornholm indicate that mink are capable of adaptation to a Danish ecosystem without competition from other wild predators. PVA may be used to evaluate population trends and the parameters affecting the population of other non-native species.

## Supporting information

S1 File (DOCX)
