# Supplementary material for: The possible impact of escaped captive American mink (Neogale vison) on the population of feral mink in Denmark
Source: PLoS One. 2023 Nov 30;18(11):e0292609. doi: 10.1371/journal.pone.0292609 (PMC10688875; doi:10.1371/journal.pone.0292609)
Supplement: S1 File — (DOCX) [file pone.0292609.s001.docx]

## S1. Appendix

## Age determination of mink

The age of mink were determined by pulp width and cementum annuli in the canine teeth. A wide pulp cavity is observed in juvenile mink when the teeth consist of >35% pulp [1]. The pulp cavity decreases when dentine is deposited inwards in the canine tooth during the animal's growth, a decrease in the size of the pulp cavity correlates with age [1,2]. Cementum annuli is used for more exact results for mink age, with one line being formed each year [3]. Age of mink was aged according to [1,4].

## Reproduction productivity of female mink

To estimate the reproduction productivity in mink, uteri were examined for scars using the staining method according to Fournier-Chambrillon et al 2010 [5]. In short, the uteri were treated with three chemical solutions; a 10% solution of ammonium-sulfide (H8N2S) for seven minutes, then washed in tap water for a few minutes, followed by immersing the uteri in a solution of equal parts 1% HCl and 20% potassium hexacyanoferrate (K4[Fe(CN)6], 3H2O), and eventually washed in tap water for a few minutes [6]. Following this process, the macrophages filled with hemosiderine were colored a black-blueish color. After this process the uteri were cut along the fallopian tubes, and the stained scars were counted and photo documented. This procedure was done only on adult female mink (>1 year) killed from April-November as the scars disappear approximately seven months after giving birth [5]. Average parturition date for mink is expected to be around April [5].

Table S1: The demographic parameters used in VORTEX to run the simulations of the mink populations.

| Scenario settings Supplementation |  |
| --- | --- |
| No. of years | 100 |
| No. of iterations | 100 |
| Duration of each year in days | 365 |
| Extinction definition | One sex remains |
| Number of populations | 1 |
| Lethal equivalents | 6.29 |
| % due to recessive lethals | 50 |
| EV concordance of reproduction & survival | 0.5 |
| No. Of catastrophes | 0 |
| Reproductive system | Polygynous |
| Age of first offspring for females | 1 |
| Age of first offspring for males | 1 |
| Maximum number of progeny per year | 11 |
| Maximum number of broods per year | 1 |
| Maximum breeding age | 5 |
| Sex ratio | 50:50 |
| % adult female breeding | Jutland = 33%, Zealand = 42%, Bornholm = 50% |
| Specify the distribution of number of offspring per female per  brood | Normal distribution |
| Fecundity | Jutland = 2, Zealand =1.9, Bornholm = 2.9 |
| Standard deviation | Jutland = 2.9, Zealand = 2.1, Bornholm = 1.9 |
| Mortality rate age 0-1 | Jutland = 24%, Zealand = 38%, Bornholm = 46% |
| Annual mortality rate after year 1 | Jutland = 40%, Zealand = 37%, Bornholm =30% |
| % male in breeding pool | 33% |
| Recent initial population size | Jutland = 266, Zealand = 273, Bornholm =201 |
| Maximum initial population size | Jutland = 6695, Zealand = 1044, Bornholm = 282 |
| Density dependent reproduction | No |
| Specified age distribution | Yes |
| Carrying capacity | Jutland = 30000, Zealand = 7100, Bornholm = 500 |
| SD in K due to EV | 0 |
| Recent population supplemented yearly | 0, 10, 20, 40, 100  Influx Jutland (80), Zealand (82), Bornholm (40) |
| Maximum population supplemented yearly | 0, 10, 20, 40, 100  Influx Jutland (1485), Zealand (209), Bornholm (56) |
| Harvest | No |

Table S2: The different the yearly number of culled mink from all the Danish municipalities in the three years which were used to make the recent scenario and high scenarios in VORTEX, using the years with the highest Game Bag Records for each of the three Danish regions Jutland (1999 – Table A2), Zealand (2013) and Bornholm (2019)

| Year | 2021 | 2019 | 2013 |
| --- | --- | --- | --- |
| København | 12 | 9 | 30 |
| Frederiksberg | 0 | 0 | 0 |
| Ballerup | 0 | 0 | 1 |
| Brøndby | 0 | 3 | 0 |
| Dragør | 0 | 18 | 12 |
| Gentofte | 1 | 6 | 7 |
| Gladsaxe | 0 | 0 | 1 |
| Glostrup | 0 | 0 | 0 |
| Herlev | 0 | 0 | 0 |
| Albertslund | 0 | 0 | 0 |
| Hvidovre | 0 | 0 | 0 |
| Høje-Taastrup | 11 | 22 | 3 |
| Lyngby-Taarnbæk | 0 | 0 | 0 |
| Rødovre | 0 | 0 | 0 |
| Ishøj | 0 | 0 | 0 |
| Tårnby | 0 | 27 | 17 |
| Vallensbæk | 0 | 0 | 1 |
| Furesø | 6 | 0 | 16 |
| Allerød | 5 | 7 | 11 |
| Fredensborg | 2 | 7 | 66 |
| Helsingør | 8 | 7 | 92 |
| Hillerød | 25 | 31 | 46 |
| Hørsholm | 0 | 0 | 0 |
| Rudersdal | 1 | 0 | 9 |
| Egedal | 8 | 7 | 34 |
| Frederikssund | 11 | 11 | 21 |
| Greve | 6 | 0 | 8 |
| Køge | 21 | 17 | 70 |
| Halsnæs | 22 | 18 | 0 |
| Roskilde | 11 | 15 | 90 |
| Solrød | 0 | 0 | 0 |
| Gribskov | 2 | 2 | 19 |
| Odsherred | 0 | 17 | 23 |
| Holbæk | 3 | 20 | 58 |
| Faxe | 9 | 11 | 48 |
| Kalundborg | 3 | 11 | 19 |
| Ringsted | 19 | 34 | 71 |
| Slagelse | 19 | 16 | 36 |
| Stevns | 22 | 2 | 20 |
| Sorø | 5 | 38 | 23 |
| Lejre | 10 | 31 | 57 |
| Næstved | 29 | 92 | 81 |
| Vordingborg | 2 | 27 | 54 |
| **ZEALAND** | **273** | **506** | **1044** |
| **BORNHOLM** | **201** | **282** | **208** |
| Haderslev | 1 | 9 | 18 |
| Billund | 2 | 27 | 46 |
| Sønderborg | 0 | 2 | 22 |
| Tønder | 3 | 16 | 13 |
| Esbjerg | 26 | 42 | 22 |
| Fanø | 0 | 0 | 0 |
| Varde | 3 | 76 | 133 |
| Vejen | 6 | 27 | 67 |
| Aabenraa | 0 | 13 | 12 |
| Fredericia | 3 | 3 | 10 |
| Horsens | 3 | 7 | 19 |
| Kolding | 2 | 0 | 16 |
| Vejle | 19 | 43 | 71 |
| Herning | 3 | 42 | 74 |
| Holstebro | 9 | 127 | 134 |
| Lemvig | 9 | 26 | 110 |
| Stuer | 2 | 10 | 21 |
| Syddjurs | 1 | 15 | 37 |
| Norddjurs | 8 | 7 | 26 |
| Favrskov | 3 | 4 | 15 |
| Odder | 0 | 2 | 0 |
| Randers | 9 | 20 | 23 |
| Silkeborg | 1 | 17 | 47 |
| Samsø | 0 | 0 | 1 |
| Skanderborg | 2 | 2 | 7 |
| Århus | 1 | 2 | 1 |
| Ikast-Brande | 2 | 6 | 23 |
| Ringkøbing | 25 | 77 | 233 |
| Hedensted | 7 | 29 | 101 |
| Viborg | 12 | 16 | 46 |
| Skive | 17 | 29 | 35 |
| Morsø | 0 | 12 | 29 |
| Thisted | 2 | 23 | 50 |
| Brønderslev | 42 | 53 | 39 |
| Frederikshavn | 6 | 20 | 89 |
| Vesthimmerland | 8 | 26 | 40 |
| Læsø | 0 | 7 | 7 |
| Rebild | 0 | 2 | 25 |
| Mariagerfjord | 1 | 4 | 26 |
| Jammerbugt | 15 | 49 | 137 |
| Aalborg | 8 | 38 | 73 |
| Hjørring | 5 | 27 | 92 |
| **Jutland** | **266** | **957** | **1990** |

Table S3: The total number of culled mink in the three Danish regions during the different years to make the recent scenario (2021), high scenarios in Jutland (1999), Zealand (2013) and on Bornholm (2019).

| Total sum of culled mink | | | | |
| --- | --- | --- | --- | --- |
| Year | 2021 | 2019 | 2013 | 1999 |
| Bornholm | 201 | 282 | 208 | 86 |
| Zealand | 273 | 506 | 1044 | 570 |
| Jutland | 266 | 957 | 1990 | 6695 |

Table S4: Significance table for the showing p-values for a Mann-Whitney U test between initial parameters and an increase or decrease of 20% in the main demographic parameters. Where bold text indicates a significant increase or decrease in the demographic parameters.

| Parameters | Jutland P-value | Zealand P-value | Bornholm P-value |
| --- | --- | --- | --- |
| Inbreeding + 20% | 0.634 | 0.950 | 0.1659 |
| Inbreeding - 20% | 0.508 | 0.805 | 0.3237 |
| Fecundity +20% | 0.064 | 0.159 | **< 2.2e-16** |
| Fecundity -20% | 0.235 | 0.448 | **< 2.2e-16** |
| Initial population +20% | 0.567 | 0.821 | **0.0238** |
| Initial population -20% | 0.344 | 0.855 | 0.1891 |
| %Breeding females +20% | 0.703 | 0.288 | **< 2.2e-16** |
| %Breeding females -20% | 0.141 | 0.791 | **< 2.2e-16** |
| Mortality +20% | 0.166 | 0.183 | **< 2.2e-16** |
| Mortality -20% | 0.337 | 0.065 | **< 2.2e-16** |

Table S5: Significance table showing p-values for a Mann-Whitney U test between initial parameters and an increase or decrease of 30% in the main demographic parameters. Where bold text indicates a significant increase or decrease in the demographic parameters.

| Parameters | Jutland P-value | | Zealand P-value | Bornholm P-value |
| --- | --- | --- | --- | --- |
| Inbreeding + 30% | 0.445 | 0.698 | | **0.02822** |
| Inbreeding - 30% | 0.849 | 0.622 | | **0.0006414** |
| Fecundity +30% | 0.211 | **0.014** | | **< 2.2e-16** |
| Fecundity -30% | 0.203 | 0.622 | | **< 2.2e-16** |
| Initial population +30% | 0.828 | 0.398 | | **0.03765** |
| Initial population -30% | 0.643 | 0.897 | | **0.001225** |
| %Breeding females +30% | **0.025** | **0.046** | | **< 2.2e-16** |
| %Breeding females -30% | 0.154 | 0.605 | | **< 2.2e-16** |
| Mortality +30% | 0.078 | 0.159 | | **< 2.2e-16** |
| Mortality -30% | **0.046** | **2.99E-06** | | **< 2.2e-16** |

## References

1. Pagh S, Pertoldi C, Chriel M, Petersen H. H, Jensen T. H, Madsen S, Kraft D. C. E, Shou T. M, Hansen M. S. Estimation of the age and reproductive performance of wild-born and escaped mink (*Neovison vison*) caught in the wild in Denmark. Animals. 11(162) 2021, 1-11.
2. Helldin J. Age determination of Eurasia pine martens by radiographs of teeth in situ. Wildlife Society Bulletin. 25(1) 1997, 83-88.
3. Grue H, Jensen B. Review of the formation of incremental lines in tooth cementum of terrestrial mammals. Danish review of game biology, 11(3) 1979, 3-44.
4. Roulichová J. Simple method of age determination in red fox, Vulpes vulpes. Folia Zool 2007, 56:440–444
5. Fournier-Chambrillon C, Bifolchi A, Mazzola-Rossi E, Sourice S, Albaret M, Bray Y, Cena J.C, Urra-Maya F, Agraffel T, Fournier P. Reliability of stained placental scar counts in farmed American mink and application to free-ranging mustelids. Journal of Mammalogy, 91(4) 2010. 818-826.
6. Bray, Y; Marboutin, E, Péroux, R, Ferron J, Reliability of stained placental-scar count in European hares. Wildlife Society Bulletin. 31(1) 2003. 237-246.
